# Efficacy and Safety of Iclaprim for the Treatment of Skin Structures and Soft Tissue Infections: A Methodological Framework

**DOI:** 10.3389/fphar.2022.932688

**Published:** 2022-07-19

**Authors:** Lian Wang, Jin Fan, Linli Zheng, Lingmin Chen

**Affiliations:** ^1^ Department of Respiratory and Critical Care Medicine, West China Hospital/West China School of Medicine, Sichuan University, Chengdu, China; ^2^ School of Health Preservation and Rehabilitation, Chengdu University of Traditional Chinese Medicine, Chengdu, China; ^3^ Mental Health Center, West China Hospital, Sichuan University, Chengdu, China; ^4^ Department of Anesthesiology and National Clinical Research Center for Geriatrics, The Research Units of West China (2018RU012, Chinese Academy of Medical Sciences), West China Hospital, Sichuan University, Chengdu, China

**Keywords:** iclaprim, skin and skin structures infections, efficacy, safety, methodology framework

## Abstract

**Background:** Skin and soft tissue infections (SSTIs) are among the most common infections worldwide. They manifest in a variety of forms, such as erysipelas, cellulitis, and necrotizing fasciitis. Antibiotics are the significant method for clinical treatment of SSTIs. This study reported a methodology framework to determine the efficacy and safety of iclaprim in treatment of SSTIs.

**Methods:** We will search the PubMed, EMbase, CNKI, WanFang Data, VIP, and ClinicalTrials.gov from their inception to June 2022 for randomized controlled trials and cohort studies on iclaprim with SSTIs. Two authors will independently screen the eligible studies, assess the quality of the included papers, and extract the required information. Randomized controlled trials will be assessed using the Cochrane risk-of-bias tool. The Newcastle–Ottawa Scale will be used to evaluate observational studies. The quality of the evidence will be evaluated using the Grading of Recommendations Assessment Development and Evaluation system. RevMan 5.3 will be used for the data synthesis and quantitative analysis.

**Results and Discussions:** This study will provide the clinicians with more high-quality evidence to choose iclaprim for patients with SSTIs.

**Ethics and Dissemination:** This systematic review and meta-analysis will be based on published data, so ethical approval is not necessary. The results of this meta-analysis will be published in a peer-reviewed journal.

## 1 Introduction

Skin and soft tissue infections (SSTIs) or skin and skin structure infections (SSSIs) have been a threatening challenge worldwide. They manifest in a variety of forms, such as impetigo, ecthyma, erysipelas, cellulitis, necrotizing fasciitis, and surgical site infections (Olaniyi et al., 2017). In the United States, the overall incidence of SSTIs increased by 40% from 2.4 million in 2000 to 3.3 million in 2012 (Lee et al., 2015). *S. aureus* is one of the most common causes of SSTIs, and it causes mild skin abscesses, superficial tissue infections, and even life-threatening diseases (Bouvet et al., 2017; Jin et al., 2021). *S. aureus* biofilm often exacerbates SSTIs and minimizes therapeutic drug activities and enhances colonization, leading to antibiotic resistance that limits treatment options (Kwiecinski et al., 2015; Olaniyi et al., 2017). The incidence of SSTIs has been on the rise, which is caused by methicillin-resistant *S. aureus* (MRSA) in hospitals and community setting (Russo et al., 2016). When the risk of exposure to MRSA is increased, the morbidity and mortality rates also increase (Miller et al., 2015). Infectious Diseases Society of America (IDSA) recommended the treatment approaches for SSTIs, which included draining, debridement, cultured and appropriate empiric antibiotic therapy (Stevens et al., 2014). The glycopeptide vancomycin has been recommended by IDSA guidelines to treat the MRSA infections. The prevalence of MRSA infection has increased dramatically in recent years (Chambers, 2001; Johnson et al., 2007), leading to the widespread use of vancomycin and the emergence of vancomycin resistance (Cosgrove et al., 2004). The emergence of MRSA and other resistant pathogens has brought a considerable challenge to its treatment. Therefore, it is of utmost importance to develop safe and effective antibiotics for the treatment of SSTIs.

Iclaprim is a diaminopyrimidine antibiotic, which potently and selectively inhibits bacterial dihydrofolate reductase and is active against Gram-positive pathogens (Schneider et al., 2003; Sader et al., 2009). Iclaprim is in the same class as trimethoprim and was designed to be more active than trimethoprim and to overcome trimethoprim resistance among Gram-positive pathogens without the need for combination with sulfonamide (Oefner et al., 2009). Previous studies have reported that iclaprim is active *in vitro* against major Gram-positive pathogens, including methicillin-susceptible *S. aureus*, MRSA, and vancomycin-resistant strains (Tenover et al., 2004; Appelbaum and Jacobs, 2005; Oefner et al., 2009). Moreover, iclaprim also shows activity against the Gram-negative respiratory pathogens *Haemophilus influenzae, Moraxella catarrhalis,* and *Chlamydia pneumoniae* (Noviello et al., 2018). In 2015, based on its potential utility, the Food and Drug Administration approved iclaprim for the treatment of acute bacterial SSSIs (Huang and Dryden, 2018). Iclaprim is a promising agent in the treatment of SSTIs. In recent years, new trials have been carried out. However, their findings were conflicted, and the efficacy and safety of iclaprim in the treatment of SSTIs remains controversial (Stevens et al., 2007; Dryden et al., 2008; Krievins et al., 2009; Huang et al., 2018a; Holland et al., 2018). Thus, we plan to conduct a meta-analysis to evaluate the efficacy and safety of iclaprim for the treatment of SSTIs.

## 2 Methods

### 2.1 Study Design

The methodology has been prepared according to the Preferred Reporting Items for Systematic Review and Meta-analysis Protocols (PRISMA-P) (Shamseer et al., 2015). This study has been registered in PROSPERO (Registration number CRD42018107278).

**FIGURE 1:**
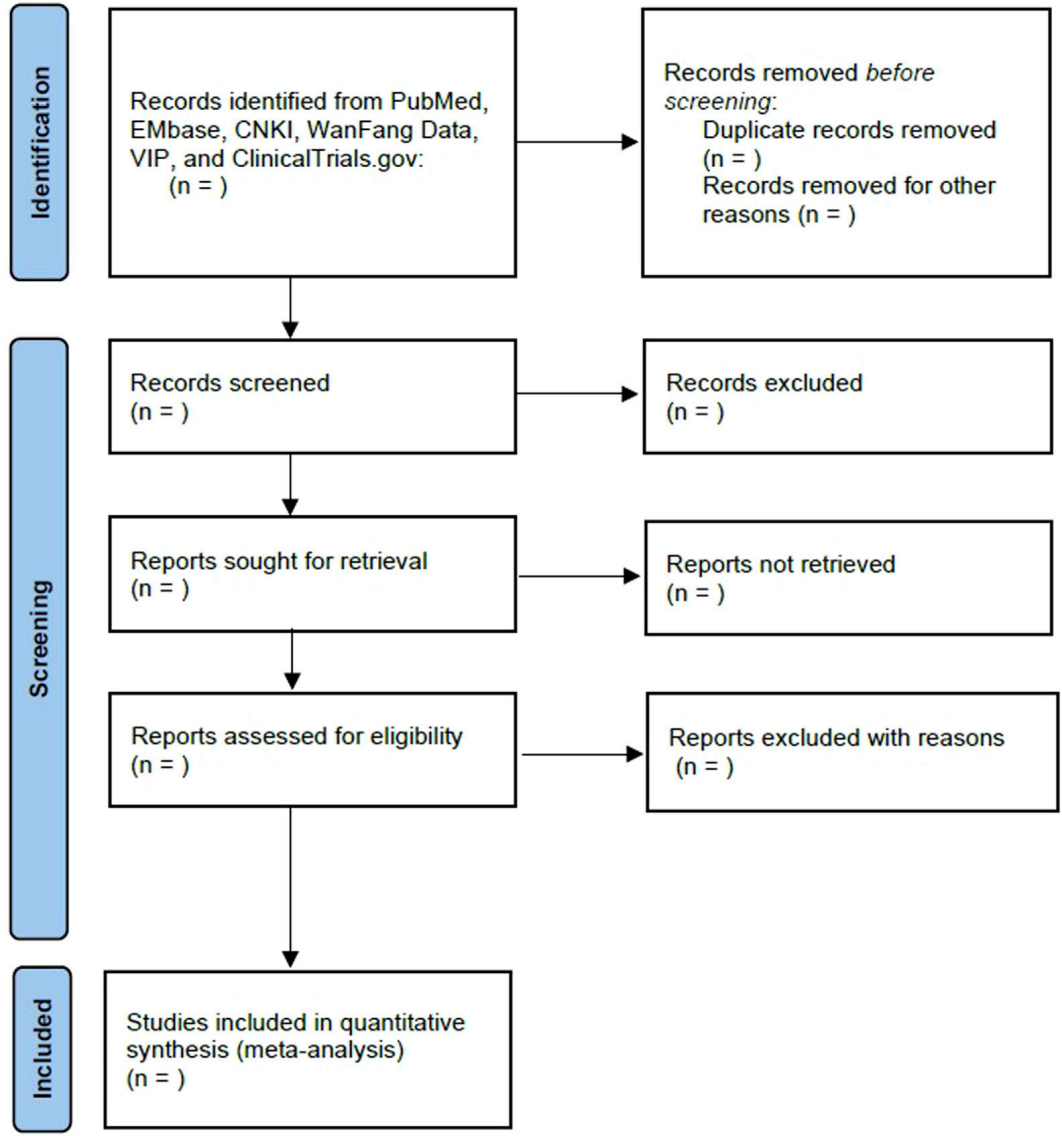
Flow diagram of the study selection process.

The future full review will be reported according to PRISMA 2020 (Page et al., 2021) (Figure 1). The current manuscript was reported as in previous studies (Liu et al., 2022; Page et al., 2021).

### 2.2 Eligibility Criteria

#### 2.2.1 Types of Studies

Relevant randomized controlled trials and cohort studies assessing the clinical efficacy and safety of lclaprim for the treatment of skin structures and soft tissue infections in patients will be included (Liu et al., 2022; Page et al., 2021). Animal studies, case reports, and case series will be excluded.

#### 2.2.2 Types of Participants

Patients aged ≥18 years with confirmed complicated SSTIs due to a Gram-positive pathogen, regardless of sex or ethnicity, will be included. SSTIs will be defined as several microbial invasions of the skin layers and of the underlying soft tissues, inducing a host response, including at least three of the following signs and symptoms: discharge, erythema, swelling and/or induration, heat and/or localized warmth, and/or pain and/or tenderness to palpation (Tognetti et al., 2012; Noviello et al., 2020). SSTIs will be sampled for microbiological culture and confirmed infections due to Gram-positive pathogens.

#### 2.2.3 Types of Interventions and Comparators

The intervention will be intravenous iclaprim with the purpose of anti-infection therapy compared with vancomycin or other antibiotics.

#### 2.2.4 Types of Outcomes

The primary outcomes will be early clinical response (ECR), clinical cure rate, and microbiologically negative rate (Krievins et al., 2009; Huang et al., 2018a; Huang and Dryden, 2018). ECR will be defined as 20% reduction in the area of the skin infection from baseline to 48–72 h after initiating antibiotics (Huang et al., 2018a). Clinical cure will be defined as the resolution of the symptoms and signs of infection and no use of any new antibiotics (Huang and Dryden, 2018). Microbiologically negative will be defined as negative culture from skin or wound at the end of the treatment (Krievins et al., 2009). The secondary outcomes will be the incidence of adverse events related to drug treatments, such as nausea, headache, diarrhea, and fatigue.

### 2.3 Search Strategy

We will systematically search the PubMed, EMbase, China National Knowledge Infrastructure (CNKI), WanFang Data, Chinese Scientific Journal Database (VIP), and ClinicalTrials.gov from their inception to June 2022, without any language restrictions (Page et al., 2021; Liu et al., 2022). Table 1 lists the search strategy of PubMed, and similar strategies will be applied to the other resources.

**TABLE 1:** PubMed search strategy.

Number	Terms
#1	“Iclaprim” (Title/Abstract)
#2	“AR 100” (Title/Abstract)
#3	“AR-100” (Title/Abstract)
#4	#1 OR #2 OR #3
#5	Skin infection (Title/Abstract)
#6	Skin structure infection (Title/Abstract)
#7	Soft tissue infection (Title/Abstract)
#8	#5 OR #6 OR #7
#9	#4 AND #8

### 2.4 Data Collection

#### 2.4.1 Study Selection

After removing duplicates, two independent authors (Jin Fan and Lian Wang) will screen and cross-check the literature according to predefined criteria, and a third author (Lingmin Chen) will reconcile any discrepancies. The selection process will follow the PRISMA 2020 flow diagram (Figure 1) (Page et al., 2021).

#### 2.4.2 Data Extraction

The following data will be gathered by two authors independently (Jin Fan and Lian Wang): (1)) basic information of study (year of publication, the name of the first author, type of study, and sample size); (2)) participant characteristics (age, sex); (3)) interventions and controls (detailed usage of iclaprim and other antibiotics); and (4)) outcomes and adverse events. Any disagreements will be discussed and judged by a third author (Lingmin Chen or Linli Zheng). We will contact the corresponding authors via email or other methods in case of missing or incorrect data (Page et al., 2021; Liu et al., 2022). If there is no response, incomplete literature will be excluded.

#### 2.4.3 Quality Assessments of Individual Studies

Randomized controlled trials will be assessed for evidence of bias with the Cochrane risk-of-bias tool (Page et al., 2021; Liu et al., 2022). The Newcastle–Ottawa Scale will be used to assess methodological strength in observational studies (Page et al., 2021; Lemma et al., 2022). Two independent authors (Jin Fan and Lian Wang) will summarize the assessments and categorize the included studies.

### 2.5 Statistical Analysis

#### 2.5.1 Meta-Analysis

This study will use RevMan 5.3 software to perform the meta-analysis (Page et al., 2021; Liu et al., 2022). Dichotomous data will be summarized using the ORs with 95% confidence intervals (CIs). Continuous outcomes will be measured using the standard mean difference or mean difference with 95% CIs. Clinical heterogeneity among studies will be assessed qualitatively, and statistical heterogeneity will be calculated using the *I* ^*2*^ measure (Page et al., 2021; Liu et al., 2022). In instances with high levels of heterogeneity (*I* ^*2*^ > 50%) among the studies, a random-effects model will be applied; otherwise, a fixed-effects model will be employed (Page et al., 2021; Liu et al., 2022).

#### 2.5.2 Publication Bias

Publication bias will be assessed by visual inspection of funnel plots (Page et al., 2021; Liu et al., 2022).

#### 2.5.3 Subgroup and Sensitivity Analysis

Subgroups analysis will be performed when meta-analysis shows significant heterogeneity and data are sufficient, such as age, treatment outcome, and type of pathogen. We plan to perform a sensitivity analysis to exclude trials of low-quality or high bias risks.

#### 2.5.4 Confidence in the Cumulative Evidence

Two authors (Lian Wang and Jin Fan) will use the Grading of Recommendations, Assessment, Development and Evaluation (GRADE) system to assess the quality of evidence associated with specific outcomes (Page et al., 2021). GRADE provides explicit criteria for rating the quality of evidence that include study design, risk of bias, imprecision, inconsistency, indirectness, and magnitude of effect (Guyatt et al., 2011). The levels of evidence will be categorized as high, moderate, low, or very low.

#### 2.5.5 Ethics and Dissemination

Ethical approval will not be necessary because this systematic review and meta-analysis only evaluated the published literature (Kim et al., 2022).

## 3 Discussion

Bacterial skin and soft tissue infections (SSTIs) are one of the most common causes of infection in patients of all ages, accounting for a large proportion of hospitalizations and emergency departments (Stevens et al., 2014). Currently, SSTIs have placed an increasing burden on healthcare systems (Hersh et al., 2008). *S. aureus* is one of the most common causes of SSTIs, especially in wound infections, abscesses, and cellulitis (Tognetti et al., 2012). Antibiotics are generally used in the clinical treatment of SSTIs. There are many antibiotics currently approved to treat SSTIs, but almost all have safety concerns or reports of drug-resistant pathogens (Huang et al., 2019).

Iclaprim is a novel diaminopyrimidine antibiotic that inhibits bacterial dihydrofolate reductase, and it is active against Gram-positive pathogens, including emerging drug-resistant pathogens, such as MRSA and vancomycin-resistant strains (Huang et al., 2020). In two randomized, double-blind phase 3 studies (ASSIST-1 and ASSIST-2) iclaprim and linezolid were compared in patients with complicated SSSI, and the pooled clinical cure rates were 82.2% (411/500) for iclaprim and 85.3% (419/491) for linezolid (Stevens et al., 2007; Dryden et al., 2008; Huang and Dryden, 2018). Besides, two randomized, double-blind, active-controlled Phase 3 studies (REVIVE-1 and REVIVE-2) were conducted to compare iclaprim and vancomycin in patients with acute bacterial skin and skin structure infections (ABSSSI). The pooled ECR was 79.6% for iclaprim and 78.8% for vancomycin, thereby showing that iclaprim achieved noninferiority (10% margin) compared with vancomycin in the treatment of ABSSSI (Huang et al., 2018a; Huang et al., 2018b; Holland et al., 2018). In clinical trials, adverse events of iclaprim were mainly nausea, diarrhea, and headache (Sincak and Schmidt, 2009). Current research shows that the incidence of vancomycin-associated acute kidney injury (AKI) ranges from 5% to 42% (van Hal et al., 2013). Replacement of vancomycin with iclaprim for the treatment of ABSSSI may avoid vancomycin-associated AKI. Therefore, we plan to conduct a meta-analysis to evaluate the efficacy and safety of iclaprim for the treatment of SSTIs. In order to include more studies, we will include randomized control trials and cohort studies, and that will be a limitation of the future meta-analysis. In addition, we will not limit the control group. However, we will perform a subgroup analysis and discuss the clinical heterogeneity in the full review. We hope this study can provide the clinicians with more high-quality evidence to choose iclaprim for patients with skin and soft tissue infections.

## Data Availability

The original contributions presented in the study are included in the article. Further inquiries can be directed to the corresponding author.
